# Re-lectotypification of *Shivparvatia glanduligera*, the type of the genus *Shivparvatia* (Alsineae, Caryophyllaceae)

**DOI:** 10.3897/phytokeys.166.58710

**Published:** 2020-11-13

**Authors:** Bine Xue, Long-Yuan Wang, Gang Yao

**Affiliations:** 1 College of Horticulture and Landscape Architecture, Zhongkai University of Agriculture and Engineering, Guangzhou 510225, China Zhongkai University of Agriculture and Engineering Guangzhou China; 2 College of Forestry and Landscape Architecture, South China Agricultural University, Guangzhou 510642, China South China Agricultural University Guangzhou China

**Keywords:** *
Arenaria
*, nomenclature, taxonomy

## Abstract

The genus *Shivparvatia* Pusalkar & D.K. Singh was described in 2015 and the specimen *J.D. Hooker 11* deposited in K (K000742193) was designated as the lectotype of its type species *S. glanduligera* (Edgew.) Pusalkar & D.K. Singh (≡ *Arenaria glanduligera* Edgew.). Nevertheless, *J.D. Hooker 11* (K000742193) is actually the type of *S. ciliolata* (Edgew.) Pusalkar & D.K. Singh (≡ *Arenaria ciliolata* Edgew.). Thus the lectotypification of *S. glanduligera* was problematic and a re-lectotypification for this species is needed. One of the syntypes of *S. glanduligera* collected from Kashmir (*H. Falconer s.n.*, K000742189) is selected here as its lectotype. Morphologically, *S. ciliolata* and *S. glanduligera* can be easily distinguished from each other by their different morphology of indumentum, disc gland and sepals, as well as the color of petals.

The genus *Arenaria* L. s.l. includes over 300 species of herbs widely distributed from Northern Temperate to arctic regions (McNeill 1962; Wu et al. 2001). It was divided into ten subgenera traditionally based on the morphology of sepals and petals, as well as the number of styles and lobes at the top of capsules (McNeill 1962). A series of recent molecular phylogenetic studies have made great progress in clarifying the circumscription of *Arenaria* s.l. (Harbaugh et al. 2010; Greenberg and Donoghue 2011; Sadeghian et al. 2015), and resulted in the disintegration of this large genus and some of its subgenera or sections were elevated as independent genera (Pusalkar and Singh 2015; Sadeghian et al. 2015).

Based on phylogenetic results, Sadeghian et al. (2015) raised the subgenus Solitaria McNeill of *Arenaria* s.l. to generic rank and described it as a new genus *Solitaria* (McNeill) Sadeghian & Zarre, with three species of subgenus Solitaria sampled in their study transferred to this new genus, viz. *Solitaria ciliolata* (Edgew.) Sadeghian & Zarre, *S. glanduligera* (Edgew.) Sadeghian & Zarre, and *S. stracheyi* (Edgew.) Sadeghian & Zarre. They also lectotypified these species except *S. glanduligera*, but three syntypes of its basionym, viz. *Arenaria glanduligera* Edgew., were cited.

On the other hand, Pusalkar and Singh (2015) elevated the subgenus Solitaria to be a new genus and described it as *Shivparvatia* Pusalkar & D.K. Singh three months earlier than Sadeghian et al. (2015). Thus the generic name *Shivparvatia* has priority in taxonomy. Pusalkar and Singh (2015) also transferred the three species of the subgenus Solitaria to their new genus, viz. *Shivparvatia ciliolata* (Edgew.) Pusalkar & D.K. Singh, *S. glanduligera* (Edgew.) Pusalkar & D.K. Singh, *S. stracheyi* (Edgew.) Pusalkar & D.K. Singh. They further designated the species *S. glanduligera* as the type of *Shivparvatia* and lectotypified *S. glanduligera*. However, they didn’t lectotypify the other two species.

In Pusalkar and Singh (2015), the Indian specimen *J.D. Hooker 11* deposited in K (K000742193, Fig. 1A) was designated as the lectotype of *Shivparvatia glanduligera*, and the other three specimens labeled as GH00353887, K000742194 (Fig. 1C) and K000742195 (Fig. 1B) were cited as isolectotypes of the species. After careful examination of the above mentioned specimens and relevant literature, we found that the four specimens cited in Pusalkar and Singh (2015) all represented *S. ciliolata* rather than *S. glanduligera*. The collection *J.D. Hooker 11* was actually the type of *S. ciliolata* (Edgeworth & Hooker, 1874), and the two specimens K000742194 and GH00353887 had been designated as the lectotype and isolectotype of *S. ciliolata*, respectively, by Sadeghian et al. (2015). The last specimen K000742195 (*J.F. Duthie 2760*, Fig. 1B) collected from Kumaun actually doesn’t share the same collector, collection number and locality with the other three specimens cited in Pusalkar and Singh (2015).

**Figure 1.**
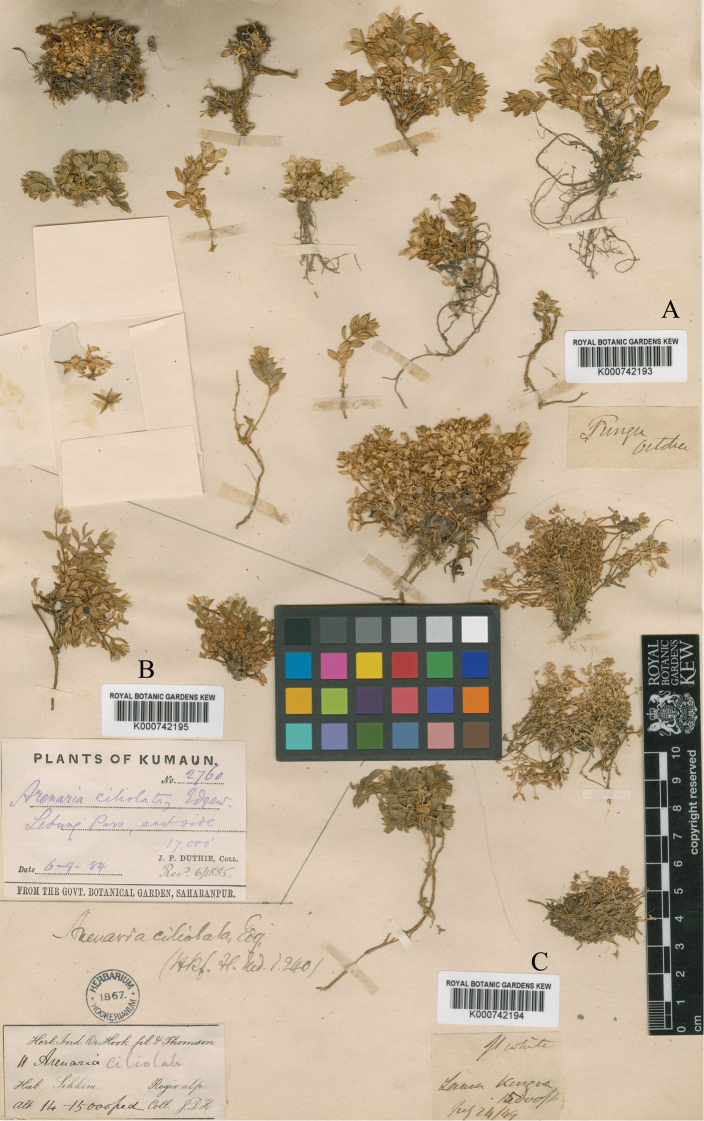
Specimens of *Solitaria ciliolata* (Edgew.) Sadeghian & Zarre **A** Isolectotype of *S. ciliolata* (*J.D. Hooker 11*, K000742193) **B***J.E. Duthie 2760* (K000742195) **C** lectotype of *S. ciliolata* (*J.D. Hooker 11*, K000742194; designated by Sadeghian et al. 2015).

It is therefore obvious that the lectotypification of *Shivparvatia glanduligera* by Pusalkar and Singh (2015) is problematic, thus a re-lectotypification of this species should be conducted. The basionym of *S. glanduligera*, viz. *Arenaria glanduligera*, was published based on three specimens: *H. Falconer s.n.* (K000742189, Fig. 2); *J.D. Hooker s.n.* (K, Fig. 3); *R. Strachey & J.E. Winterbottom s.n.* (K, Fig. 4) (Edgeworth and Hooker 1874). We therefore reselected one specimen from the syntypes of *S. glanduligera* as its lectotype. As only the specimen *H. Falconer s.n.* has been assigned a barcode number (K000742189), it is therefore selected.

## Typification

### Shivparvatia glanduligera

Taxon classificationPlantaeCaryophyllalesCaryophyllaceae

(Edgew.) Pusalkar & D.K. Singh, J. Jpn. Bot. 90: 84. 2015

34818813-D3B8-589C-BAB6-E3155FF39213

Figs 2 , 3 , 4

≡ Arenaria glanduligera Edgew. in Edgew. & Hook. f., Fl. Brit. India 1: 240. 1874.

#### Type.

**Figure 2.**
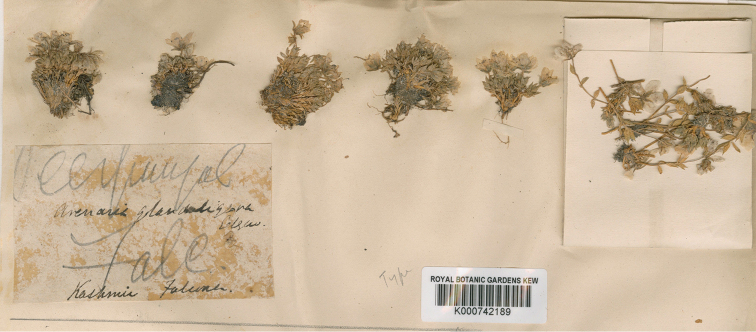
Lecotype of *Shivparvatia glanduligera* (Edgew.) Pusalkar & D.K. Singh from Kashmir (*H. Falconer s.n.*, K000742189).

**Figure 3.**
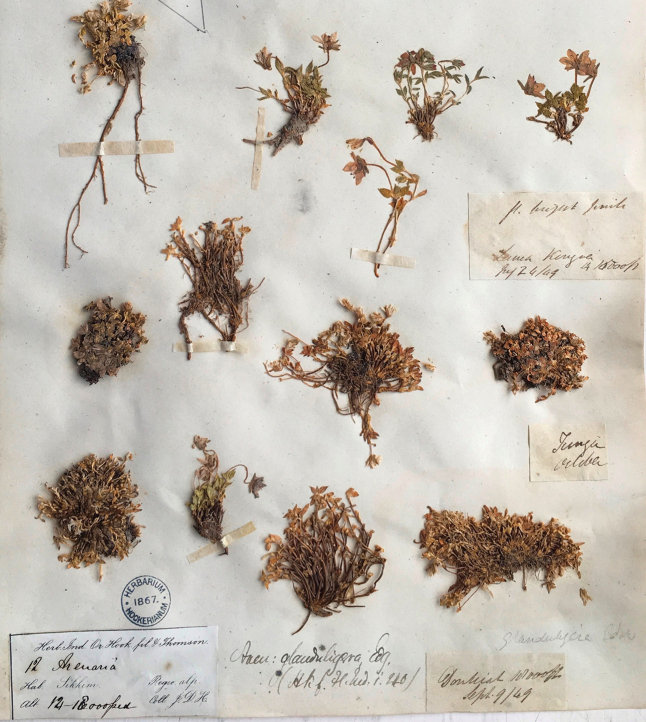
Syntype of *Shivparvatia glanduligera* (Edgew.) Pusalkar & D.K. Singh from Sikkim (*J.D. Hooker s.n.*, K).

**Figure 4.**
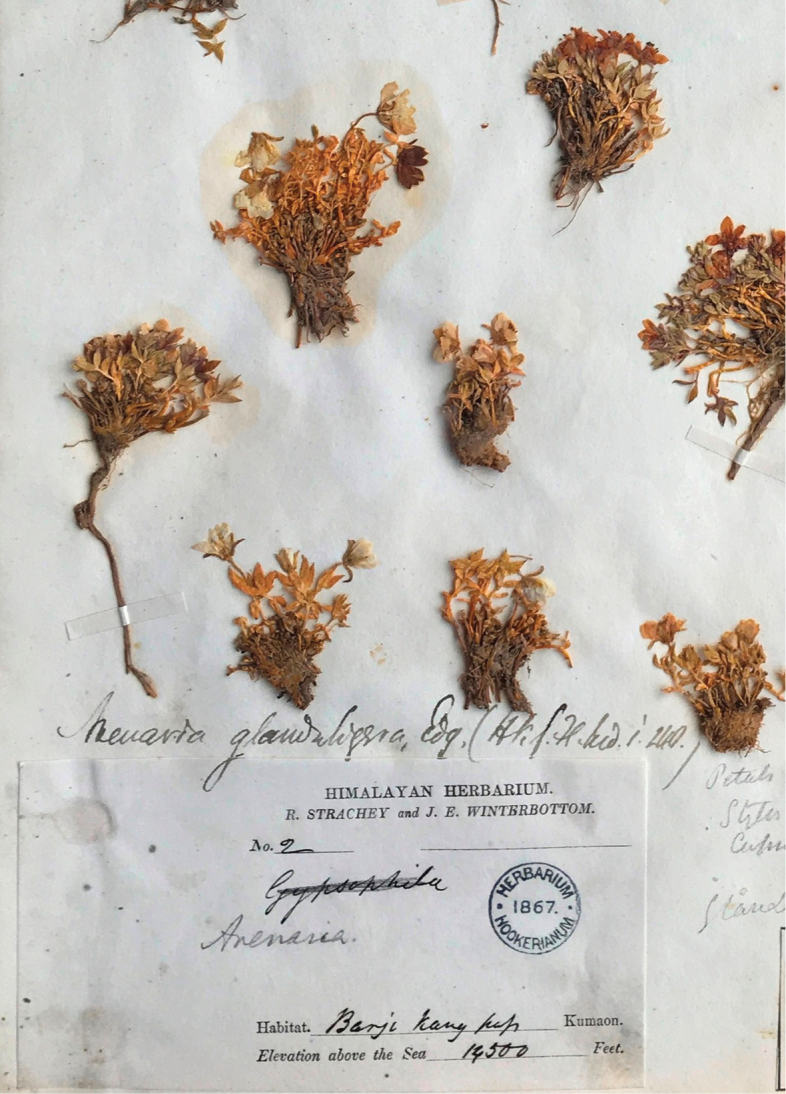
Syntype of *Shivparvatia glanduligera* (Edgew.) Pusalkar & D.K. Singh from Kumaon (*R. Strachey & J.E. Winterbottom s.n.*, K).

***Lectotype*** (designated here): Kashmir, *H. Falconer s.n.* (K000742189, Fig. 2); Remaining syntypes: INDIA. Interior of Sikkim, 14,000‒18,000 ft, *J.D. Hooker s.n.* (K, Fig. 3); Kumaon, Barji Kang pass, 14,500 ft, *R. Strachey & J.E. Winterbottom s.n.* (K, Fig. 4).

#### Note.

Morphologically, *S. glanduligera* can be easily distinguished from *S. ciliolata* by its glandular pubescence, prominent disc gland, not pure white and usually violet petals, and scarious sepal margin. In contrast, the latter is characterized by its ciliate pubescence, small disc gland, entirely pure white petals, and thickened sepal margin (Wu et al. 2001; Pusalkar and Singh 2015).

## Supplementary Material

XML Treatment for Shivparvatia glanduligera
